# Multisystemic Inflammatory Syndrome in Children, A Disease with Too Many Faces: A Single-Center Experience

**DOI:** 10.3390/jcm11185256

**Published:** 2022-09-06

**Authors:** Alina Grama, Simona Sorana Căinap, Alexandra Mititelu, Cristina Blag, Claudia Simu, Lucia Burac, Bianca Simionescu, Camelia Mărgescu, Genel Sur, Mihaela Spârchez, Mădălina Bota, Beatrice Tănasă, Tudor Lucian Pop

**Affiliations:** 1 Pediatric Discipline, Department of Mother and Child, Iuliu Hațieganu University of Medicine and Pharmacy, 400177 Cluj-Napoca, Romania; 2 Pediatric Clinic, Emergency Clinical Hospital for Children, 400177 Cluj-Napoca, Romania

**Keywords:** multisystemic inflammatory syndrome, children, SARS-CoV-2, infection, evolution

## Abstract

Background and aim: Multisystemic inflammatory syndrome in children (MIS-C) is a rare and severe condition associated with Severe Acute Respiratory Syndrome Coronavirus (SARS-CoV-2) infection in children with onset approximately 4–6 weeks after infection. To date, the precise mechanism that causes MIS-C is not known and there are many questions related to the etiology, risk factors, and evolution of this syndrome. We aimed to describe the clinical manifestations, treatment methods, and disease evolution and analyze the main risk factors for MIS-C in children hospitalized in our clinic. Material and methods: We performed a retrospective study including children with MIS-C followed-up in the 2nd Pediatric Clinic of the Emergency Clinical Hospital for Children Cluj-Napoca, Romania, for 13 months (November 2020–December 2021). Results: We included in our cohort 34 children (mean age 6.8 ± 4.6 years) who met MIS-C criteria: high and prolonged fever associated with organ dysfunction (heart, lungs, kidneys, brain, skin, eyes, bone marrow or gastrointestinal organs), and autoantibodies and/or polymerase chain reaction positives for SARS-CoV-2. Nineteen patients (55.88%) had a severe form of the disease, with multiorgan failure and shock, and myocardial or respiratory failure. The number of organs affected in the severe forms was significantly higher (more than 6 in 73.70%) than in mild forms (2–3 in 60%). Cardiac dysfunction, hypoalbuminemia, hypertriglyceridemia and hyponatremia were more important in severe forms of MIS-C. These patients required respiratory support, resuscitation with fluid boluses, vasoactive drugs, or aggressive therapy. All patients with mild forms had fully recovered compared to 63.16% in severe forms. The others with severe forms developed long-term complications (dilation of the coronary arteries, premature ventricular contraction, or myocardial fibrosis). Two patients had an extremely severe evolution. One is still waiting for a heart transplant, and the other died (hemophagocytic lymphohistiocytosis syndrome with multiorgan failure). Conclusions: From mild to severe forms with multiorgan failure, shock, and many other complications, MIS-C represents a difficult challenge for pediatricians, who must be aware of the correct diagnosis and unpredictable, possibly severe evolution.

## 1. Introduction

Since December 2019, when the first cases appeared in Wuhan, China, Severe Acute Respiratory Syndrome Coronavirus (SARS-CoV-2) has spread rapidly throughout the world, affecting people of all ages, from neonates to the elderly. Children represent only 2–5% of the population diagnosed with Coronavirus infection (COVID-19), which ranges from asymptomatic infections to critical illnesses [1,2]. The most severe late complication of this infection in the pediatric population is multisystemic inflammatory syndrome in children (MIS-C), also named pediatric inflammatory multisystem syndrome (PIMS). MIS-C was described for the first time in April 2020, and it is characterized by a state of hyperinflammation with multiorgan damage, shock, and features compatible with Kawasaki disease (KD) or toxic shock syndrome [2]. The clinical manifestations can be more severe than acute COVID-19, requiring mandatory hospitalization and sometimes intensive care measures. Often, MIS-C develops in children without comorbidities, but obesity, respiratory diseases (including asthma or chronic lung disease), cardiovascular diseases, and immunodeficiencies are risk factors [1,2].

According to the Center for Disease Control and Prevention (CDC) and World Health Organization (WHO), MIS-C is defined as a syndrome that occurs in patients up to 21 years of age and is characterized by persistent fever (that lasts 24 h or longer), constantly increasing markers of inflammation such as C-reactive protein (CRP), erythrocyte sedimentation rate (ESR), D-dimers, procalcitonin, interleukin-10 (IL-10) or tumor necrosis factor (TNF), multiple organic dysfunction (heart, lungs, kidneys, brain, skin, eyes, bone marrow or gastrointestinal organs), and serologic test and/or polymerase chain reaction positive for SARS-CoV-2 [3,4,5].

There are still many unsolved questions related to the etiology, risk factors, or evolution of MIS-C. Therefore, the etiopathogenetic mechanism of MIS-C is not known precisely. It is supposed that genetic factors responsible for innate immunity alter the immune response to SARS-CoV-2 and thus may be considered a significant factor in the etiopathogenesis of MIS-C in some children [3]. MIS-C has not yet been defined as a disease and is considered a clinical–biological syndrome.

This study aimed to describe the clinical manifestations, treatment methods, and disease evolution and analyze the main risk factors for MIS-C in children hospitalized in our clinic.

## 2. Materials and Methods

We performed a retrospective study including children with MIS-C followed-up in the 2nd Pediatric Clinic of the Emergency Clinical Hospital for Children Cluj-Napoca, Romania, for 13 months, from November 2020 to December 2021. Since the virus first appeared in Wuhan in January 2020, four major strains have circulated the world, and especially the second and fourth waves were associated with MIS-C.

The inclusion criteria were children under 18 years with a diagnosis of MIS-C based on CDC and WHO criteria: fever more than 24 h with multisystem dysfunction of at least 2 organs/systems (cardiovascular, respiratory, kidney, neurological, hematological, gastrointestinal, mucocutaneous), elevated markers of inflammation (CRP, ESR, procalcitonin), no other cause of inflammation (including bacterial sepsis, other viral infections, staphylococcal toxic shock syndrome or other inflammatory disorders), and evidence of SARS-CoV-2 infection (RT-PCR positive, antigen test or SARS-CoV-2 antibodies, or direct contact with a person infected with SARS-CoV-2) [3,4,5,6,7].

Based on clinical presentation and the presence of multiorgan dysfunction or shock, we classified MIS-C into mild and severe forms of the disease. We considered as severe MIS-C the presence of respiratory failure that required oxygen therapy (through a nasal cannula, mask or mechanical ventilation), acute respiratory distress syndrome (ARDS), significant cardiac involvement with left ventricular ejection fraction (LVEF) less than 55%, coronary dilatation with z-score between 2 and 2.5 or coronary aneurysms with z-score >2.5, elevated high-sensitive cardiac troponin I (hs-cTnI, Advia Centaur, Siemens Healthcare Diagnostics Inc., Tarrytown, NY, USA) more than 2.5 ng/L or increased brain natriuretic peptide (NT-proBNP) > 125 pg/mL, signs of shock, refractory arterial hypotension with mean blood pressure under the 5th percentile of the reference value for age, and the need for vasoactive medication for signs of hypoperfusion (impaired organ perfusion such as decreased urine output, altered mental status, or delayed peripheral perfusion manifested by weak peripheral pulses, delayed capillary refill >2 sec, cool extremities) [8,9]. The presence of severe abdominal pain (appendicitis-like) or laboratory evidence of acute kidney injury (elevation in serum creatinine levels 1.5–1.9 times baseline and/or decrease in urine output <0.5 mL/kg/h for 6–12 h), acute liver failure (ALF) based on pediatric acute liver failure (PALF) criteria, hepatic coagulopathy not corrected by parenteral administration of vitamin K or active bleeding, and/or severe thrombocytopenia (platelet count < 50 × 10^3^/mm^3^) represented another inclusion criterion for severe MIS-C [10,11,12,13]. Regarding the treatment, 23 patients (67.65%) received dual therapy with IVIG (1–2 g/kg/day, max 60 mg/day) and corticosteroids (1–2 mg/kg/day). We used IVIG alone in six patients (17.65%) with KD-like presentation without shock. In five children (14.70%) with a mild form of the disease and without cardiac dysfunction or coronary abnormalities, we used only low doses of prednisolone (1–2 mg/kg/day, max 60 mg/day). According to our local protocol, all patients with KD-like, ventricular dysfunction, or coronary artery abnormalities (*n* = 17; 50%) received low doses of aspirin (3–5 mg/kg/day, max 100 g). In two patients with a very severe disease without response to initial treatment, methylprednisolone sodium succinate in pulse therapy, anakinra (5 mg/kg/day intravenous), or recombinant interleukin-6 receptor (Tocilizumab) was used. Albumin infusion was necessary for 13 patients (38.24%) with edema secondary to hypoalbuminemia. Based on the increased risk of thrombotic complications, we administered prophylactic enoxaparin in 13 patients (38.24%) according to our protocol when the D-dimer level was greater than five times the upper limit of the normal.

We excluded from the study children who did not meet the MIS-C criteria, patients with other causes of systemic inflammation (bacterial or viral infections, autoimmune disorders), patients with ambiguous diagnoses, incomplete data in the observation sheets, or those lost from the follow-up.

We analyzed the demographic characteristics (age, gender), clinical manifestations (onset symptoms, clinical exam, and complications), laboratory and imaging characteristics, the therapeutic methods, and the outcome. We also studied data related to the patient history of COVID-19. Complete blood count, hepatic enzymes, renal function tests, glycemia, serum electrolytes, inflammation, and coagulation parameters were performed in all patients. In 25 children, we measured the level of interleukin-6 (IL-6). Regardless of the onset of symptoms, all children were evaluated cardiologically (NT-proBNP or troponin level, echocardiography, electrocardiogram, Holter monitoring) at first admission to our hospital or during the follow-up. In five patients, we performed osteomedullary aspiration for hematological manifestations (pancytopenia, thrombocytopenia), and lumbar puncture was made in two children with associated neurological manifestations. After discharge, the patients were monitored initially weekly, then monthly (clinically, laboratory tests and ultrasound examination).

This study was carried out according to the Declaration of Helsinki principles after obtaining informed consent from the parents.

All data were analyzed statistically using the Statistical software, Version 13, TIBCO Software Inc. (Palo Alto, CA, USA). We used descriptive statistics for continuous variables (means and standard deviations) and categorical variables (numbers and percentages). The Student t-test was used to analyze the statistical significance of the categorical parameters with normal distribution. We used the N-1 Pearson Chi-square test for the comparison of proportions. We analyzed the statistical significance of the differences between the parameters in the mild and severe forms of MIS-C. For IL-6, we analyzed only the patients who had this test performed (25 out of 34 patients). The results were considered statistically significant at values of *p* < 0.05.

## 3. Results

Our study includes 34 children aged between 5 months and 16 years and 10 months (mean age of 6.8 ± 4.6 years). There are 21 males (61.67%). All children were Caucasian, with a predominance (58.82%) of those from the urban area. Most of the patients were previously healthy, and only a few presented comorbidities such as asthma (three patients, 8.82%), atopic dermatitis (two patients, 5.88%), or repeated episodes of upper respiratory tract infection (two patients, 5.88%). Malnutrition (weight-for-height z-score of -2.0 or lower) was present at admission in five patients (14.70%) and two patients (5.88%) were found to be overweight (BMI > 25 kg/m^2^ or >85th percentile for age/sex).

Children in our study presented high fever, fatigue, abdominal pain, rash, conjunctivitis, hematological manifestations (pancytopenia, macrophage activation syndrome), hepatitis, myocarditis, or KD manifestations. The most important symptoms and demographic characteristics in children from our cohort are presented in Table 1.

All patients had positive serology for SARS-CoV-2, and no one tested positive for RT-PCR. Only three patients recognized the time of infection with the SARS-CoV-2 about a month before, and 12 patients were in contact with SARS-CoV-2 positive people.

Gastrointestinal involvement was the most frequently reported organ dysfunction in MIS-C in the present study but was very close to cardiac and hematological involvement. Our patients often presented mild symptoms that mimicked viral gastroenteritis, dehydration, abdominal pain, vomiting, and diarrhea. In five patients (14.70%), clinical manifestations and abdominal ultrasound (US) were suggestive of acute appendicitis; two (5.88%) had surgery, unfortunately without histopathologic evidence of appendicitis. Alanine aminotransferase (ALT) and/or aspartate aminotransferase (AST) were elevated in five patients (14.70%), and one child (2.94%) developed ALF. The pancreas was involved in two patients (5.88%), showing a minimal pancreatic reaction without the criteria of acute pancreatitis. The abdominal US or magnetic resonance imaging (MRI) revealed acalculous cholecystitis in one patient (2.94%) and ascites or mesenteric adenitis in four (11.76%) other patients.

Evidence of cardiac involvement was found in 26 patients (74.47%), with tachycardia in 25 (73.52%). Moderate (LVEF < 55%) and severe decreased left ventricular ejection fraction (LVEF < 30%) have been documented in five (20.58%) and two cases (5.88%), respectively. Pericardial effusion was found in a quarter of patients (nine cases: 26.47%) without hemodynamic consequences.

Mucocutaneous manifestations (64.71%) have been various and highly polymorphic, ranging from minimal rash to features of KD, toxic shock syndrome, or necrotizing lesions. Rash, red eyes, strawberry tongue, red hands and feet, and cracked lips have been so typical that they have become a significant clinical sign of the diagnosis. Other cutaneous findings encountered in our patients were maculopapular eruptions like measles, urticarial lesions, livedo reticularis, papulovesicular, petechiae lesions, and erythema-multiforme lesions. Also, a critically ill infant developed purpura fulminans, which evolved rapidly towards skin necrosis, requiring plastic surgical treatment.

Neurological involvement was present in more than three-quarters of our patients (24 patients, 70.59%). There were often mild manifestations, with irritability, drowsiness, and headache. Two patients presented severe encephalitis characterized by headache, photophobia, drowsiness, irritability and meningism, and EEG abnormalities (slow-wave activity). Seizures were not a feature of our group, even though reported in the literature as one of the primary neurological manifestations of children with MIS-C [13].

In the group with severe evolution of MIS-C, the mean age was lower than in the mild group, and the duration of hospitalization was significantly longer (Table 2). Also, the number of fever days was almost double in those with severe impairment, and laboratory parameters showed pronounced inflammatory markers, especially CRP and procalcitonin. Heart and hematological damage were significantly more severe in the second group, confirmed by increased NT-proBNP or troponin and hematological changes, especially thrombocytopenia. Triglycerides level was also higher in the severe disease group, and serum sodium was low.

In Table 3, we compared mild and severe forms of the disease according to the number of affected organs, the severity of the tissue injuries produced, and the complications.

As expected, the number of organs affected in severe forms of MIS-C was significantly higher than in mild forms (more than six organs in 73.70% of severe forms compared to up to five organs in all mild forms). Three patients developed a severe form of the disease with shock and multiorgan dysfunction, requiring respiratory support, resuscitation with fluid boluses and vasoactive drugs. The markers of inflammation (ESR, CRP, procalcitonin, IL-6) were increased in both categories of patients, especially in severe forms (*p* < 0.05). Cardiac involvement was significantly more frequent and serious in the severe forms, confirmed not only by the high value of the cardiac parameters but also by the complications developed in the long term: coronary artery dilatation (*n* = 3; 15.79%), myocardial fibrosis (*n* = 1; 5.26%), congestive heart failure (*n* = 1; 5.26%), or premature ventricular extrasystoles (*n* = 1; 5.26%). Renal involvement was present in almost all patients regardless of the form. Liver function was altered, especially in severe forms of MIS-C, in which hypoalbuminemia determined the appearance of edema and serositis. The increase in the risk for thrombosis, highlighted by the increase in d-dimers, was more common in severe forms of MIS-C (*p* = 0.0073). Two children (5.88%) developed severe hematological complications, one macrophage activation syndrome and the other hemophagocytic lymphohistiocytosis (HLH). Despite therapy with the HLH-2004 protocol [14] (including etoposide, dexamethasone, and cyclosporine), the patient with HLH had an unfavorable evolution.

## 4. Discussion

After initial concerns about the COVID-19 pandemic, an extremely severe complication in children, MIS-C, occurs about a month after infection and becomes increasingly relevant. The few existing studies report an incidence of 2 to 5% [3]. It is unknown whether the triggers of MIS-C only in some patients after COVID-19 are related to the genetic status of the host or other biological or environmental factors. So far, the incidence of MIS-C differs depending on the geographical area, being more common in Europe and America. Other factors predisposing to this pathology are obesity and males, but existing studies are still too few to draw definitive conclusions [3,15].

In children with MIS-C, symptoms are variable and can get worse quickly; about 7% of patients develop shock or multiorgan organ failure. The child is often ill, with a fever for many days, skin rash, conjunctival congestion, and digestive, neurological, cardiac, or hematological manifestations [3].

In our study, gastrointestinal symptoms occur in over 70% of MIS-C patients, ranging from abdominal pain to acute gallbladder hydrops or mimicking acute appendicitis, which caused unnecessary laparotomies. Studies reported that digestive manifestations occur in over 80% of MIS-C cases, often mimicking infectious gastroenteritis, inflammatory bowel disease, or acute hepatitis [16,17]. Most were accompanied by systemic manifestations (fever, rash, dysfunction of other organs) and markedly elevated inflammatory markers. The mechanism of intestinal involvement in MIS-C is not known precisely. It is supposed that appendix involvement is secondary to increased expression of angiotensin-converting enzyme 2 (ACE2) receptor in the terminal ileum. Therefore, MIS-C can present characteristics of acute appendicitis without any sign of acute transmural inflammation. There are differences between real appendicitis and MIS-C, such as leucopenia and thrombocytopenia in the latter [16,17,18].

Regarding hepatobiliary involvement, it seems that ACE2 receptors are more expressed in cholangiocytes (59.7%), followed by the endothelial layer of small blood vessels and less in hepatocytes (only 2.6%) and sinusoidal endothelial cells sinusoidal [19]. Biliary tract involvement is more commonly described than direct liver damage in children with MIS-C. In our study, hepatobiliary involvement was represented by sludge in the gallbladder, thickening of the gallbladder wall, biliary hydrops, and acute hepatitis. Although quite common in our patients, direct liver injury is challenging to attribute only to the hyperinflammatory process in MIS-C. It is already known that SARS-CoV-2 infection might have destructive effects on the liver through several mechanisms: direct cytotoxicity from active viral replication of SARS-CoV-2 in the liver, immune-mediated liver damage, endothelitis or vascular impairment due to coagulopathy, vascular stasis due to cardiac congestion, hypoxic lesions secondary to respiratory failure, or drug-induced liver injury [20]. Compared to acute SARS-CoV-2 infection that can cause direct liver damage by binding to ACE2 receptors found on the surface of the liver and bile duct epithelial cells, in MIS-C, liver tissue damage appears to be immune-mediated or secondary to cytokines release, causing changes similar to sepsis-associated liver dysfunction: elevated liver markers, elevated bilirubin concentration, and impaired synthesis function resulting in hypoproteinemia and coagulation disorders [19,20,21]. Hepatocellular damage is characterized by zones of lobular apoptosis, necrosis, portal inflammation, steatosis, cholangiocellular damage, and inflammation of the bile ducts [20]. According to some authors, liver damage in MIS-C is associated with a poor prognosis, and children with hepatitis at admission present a higher risk of developing shock [22]. In our cohort, three patients who developed shock presented moderately increased transaminase levels at admission, so it is difficult to establish a correlation between liver damage and evolution. Most of our patients probably presented an increase of transaminases during hospitalization, secondary to several factors (inflammation, immune reaction, medication) and not just the cytokine storm.

A specific feature of MIS-C is the high prevalence (up to 100%) of cardiac involvement, such as myocarditis, coronary dilatation, or cardiogenic–vasoplegic shock [5,23]. It is essential to carefully monitor these patients because of the risk of developing coronary artery aneurysms [24]. In our study, the most common cardiac abnormality was left ventricular dysfunction, followed by coronary dilatation. Initially, the clinical manifestations of children with MIS-C were classified as KD, with certain common features such as prolonged fever, conjunctivitis, acute mucocutaneous inflammation, and heart damage [25]. In evolution, it becomes evident that although both diseases occur due to an exaggerated inflammatory response and some clinical manifestations overlap, they are separate entities; children with MIS-C associate severe gastrointestinal manifestations, coagulopathy, hematological symptoms, neurological symptoms, or shock [26,27]. Also, the average age of children with MIS-C is 8–9 years, whereas the average age of children with KD is 3 years, the maximum incidence being in infants [28]. Another specific feature of MIS-C that differentiates it from KD is that coronary artery dilation rarely leads to the formation of aneurysms [2,29,30,31]. Even though the evolution of these two entities is different, MIS-C mortality is 1–2%, whereas KD < 0.1% [29]. Myocardial injury in MIS-C is characterized primarily by myocardial edema associated with vasculitis lesions and less by necrosis lesions, as they occur in myocarditis secondary to other infectious causes. This explains the excellent response to prednisone therapy in MIS-C [32,33]. Furthermore, the high incidence of giant aneurysms in infants suggests that the structure of the vessels during growth might impact their mechanical resistance [34].

Hematological manifestations are common in patients with MIS-C (up to 76% of cases). These children present lymphopenia, anemia, or thrombocytopenia. In some patients, the bone marrow injury is highly severe; two of our patients developed secondary hemophagocytic lymphohistiocytosis (HLH)/macrophage activation syndrome (MAS). HLH is characterized by an uncontrolled activation and proliferation of T lymphocytes and macrophages, which causes an increased release of proinflammatory cytokines and an excessive inflammatory reaction. Five of the following criteria are needed to establish the diagnosis: fever, splenomegaly, bicytopenia, hypofibrinogenemia (<1.5 g/L) or hypertriglyceridemia, hyperferritinemia (>500 ng/mL), decreased natural killer cell activity (NK), IL-2 receptor (soluble r-CD25) >2.400 U/mL, and the presence of hemophagocytosis in the bone marrow, spleen, or liver [35]. Moreover, MIS-C is associated with an increased risk of thromboembolic events due to the procoagulant status triggered by the infection and maintained by the inflammatory process [36]. Our children with MIS-C and laboratory findings suggestive of a pro-coagulable state have benefited from the anticoagulant therapy either for curative or prophylactic purposes.

Our cohort’s skin and ophthalmological manifestations were quite common, representing the first step toward the specific investigations for MIS-C in children with prolonged fever [37,38]. Generally, mucocutaneous involvement can be highly varied, ranging from diffuse rash, as in toxic shock syndrome, to maculopapular eruptions, urticarial lesions, livedo reticularis, papulovesicular or varicella-like lesions, petechiae- or dengue-like lesions, erythema-multiforme-like lesions, skin desquamation, palmar or feet erythema/edema, cheilitis, cracked lips, strawberry tongue, etc. Mucocutaneous findings in MIS-C may be like those seen in KD. Nevertheless, these two conditions differ significantly regarding the age of onset, race predilection, clinical manifestations, or evolution [37]. Studies published last year describe skin findings as a common manifestation in MIS-C, with an incidence between 60% to 75%. Also, half of the children who met MIS-C criteria associate conjunctivitis, and more than 20% had oral mucosal changes. The presence of mucocutaneous findings was significantly associated with disease severity, these manifestations being found in most patients with severe forms of the disease [36,37]. Skin manifestation in MIS-C is often reversible after combining IVIG and corticosteroids. The administration of IVIG caused only a slight amelioration of the skin–mucous lesions, the evolution being quickly favorable after the association of steroids (1–2 days) [37,38].

Many patients from our cohort presented neurological manifestations, even if they were not severe. We noticed marked irritability in the first days of hospitalization, especially at younger ages, which improved significantly with the introduction of corticosteroid therapy. The most common manifestations were headache, marked drowsiness, or photophobia. Only two children developed encephalitis, with a favorable evolution after intravenous methylprednisolone (30 mg/kg/day for 3 days) and IVIG. Neurological manifestations in MIS-C were reported to have a favorable prognosis, often with complete recovery if specific therapy was initiated early [39]. Inflammation in the central nervous system, which includes the brain and spinal cord, has been described in up to 50% of the cases with MIS-C and seems to be due to the presence of ACE2 receptor and immune dysregulation after cytokine storms in the absence of early therapy, severe complications like cerebral artery infarction, cerebral edema, subarachnoid hemorrhage, status epilepticus, or death [12,40].

The severe forms of MIS-C predominantly involve cardiovascular, gastrointestinal, and neurological organ systems, and cardiac involvement significantly worsened the prognosis of the disease in these patients. The parameters specific to heart dysfunction (left ventricular ejection fraction, coronary dilatation, or aneurysms z-score, high-sensitive cardiac troponin T and brain natriuretic peptide) were significantly modified in severe forms of the disease, and important cardiovascular manifestations have been the basis for Pediatric Intensive Care Unit (PICU) admission. As expected, patients with a severe form of MIS-C had significantly increased markers of inflammation, which was also validated by a higher number of fever days, a higher incidence of episodes of solemn shivering, or a twice more extensive hospitalization. Based on hospitalization data, progression to severe evolution can be assessed, but extensive studies are needed.

All our patients with a mild form of MIS-C fully recovered. Unfortunately, we cannot say the same about the severe forms of the disease, in which the recovery was complete only in half of the cases. Most of them developed cardiac complications such as the dilation of the coronary arteries, premature ventricular contraction, or myocardial fibrosis. The disease course of MIS-C was also extremely severe in two teenagers, one who developed congestive heart failure and is currently on the heart transplant list and the other one who died of multiorgan failure and HLH.

## 5. Conclusions

There are currently many unknown data regarding MIS-C, including its appearance only in some children with various manifestations. From mild forms of the disease, manifested with fever, sometimes rashes, or moderate injury, to severe forms with multiorgan failure, shock, and many other complications, MIS-C represents a difficult challenge for pediatricians, mainly due to still many unknowns about this pathology. Based on our clinical experience, this study aimed to contribute to a better understanding of MIS-C, especially as the new waves of COVID-19 are coming and the pediatric community must be aware of the correct diagnosis and possibly severe evolution.

## Figures and Tables

**Table 1:** Characteristics at the first presentation of children with MIS-C.

Characteristics	(No./%)
**Mean Age (years ± SD)**	6.8 ± 4.6
**Males**	21 (61.76%)
**Urban area**	20 (58.82%)
**Caucasian**	34 (100%)
**Known SARS-CoV-2 infection**	3 (8.82%)
**Recent contact SARS-CoV-2**	12 (32.35%)
**Cardiac manifestations** tachycardia mild myocardial dysfunction (LVEF between 30–55%) severe myocardial dysfunction (LVEF less than 30%) hypotension shock with a vasopressor requirement arterial hypertension Kawasaki disease like coronary dilatation/aneurysm pericardial effusion abnormalities on echocardiogram or dysrhythmia	**26 (74.47%)** 25 (73.52%) 7 (20.58%) 2 (5.88%) 9 (26.47%) 4 (11.76%) 2 (5.88%) 3 (8.82%) 5 (14.70%) 9 (26.47%) 1 (2.94%)
**Gastrointestinal symptoms:** gastroenteritis acute hepatitis acute liver failure sludge in the gallbladder biliary hydrops false appendicitis mesenteric adenitis ascites	**27 (79.41%)** 17 (50.00%) 5 (14.70%) 1 (2.94%) 4 (11.76%) 1 (2.94%) 5 (14.70%) 4 (11.76%) 4 (11.76%)
**Neurological symptoms:** headache vision troubles marked drowsiness meningism encephalitis	**24 (70.59%)** 6 (17.64%) 2 (5.88%) 14 (41.17%) 2 (5.88%) 1 (2.94%)
**Respiratory symptoms** (pneumonia, hypoxia)	**13 (38.24%)**
**Hematological manifestations:** leukopenia thrombocytopenia anemia bicytopenia pancytopenia macrophage activation syndrome hemophagocytic lymphohistiocytosis syndrome	**26 (76.74%)** 13 (38.24%) 13 (38.23%) 18 (52.94%) 7 (20.58%) 5 (5.88%) 1 (2.94%) 1 (2.94%)
**Mucocutaneous damage:** rash peeling of skin around fingernails and toenails conjunctivitis mucositis	**22 (64.71%)** 12 (35.29%) 5 (14.70%) 21 (61.76%) 5 (14.70%)
**Acute kidney injury**	**7 (20.59%)**
**Rheumatic symptoms** arthralgia/arthritis myalgia	**13 (38.24%)** 3 (8.82%) 6 (17.64%)
**Other symptoms:** fever shivering asthenia edema	34 (100%) 8 (23.53%) 29 (85.29) 10 (29.41%)
**Children admitted to the Pediatric Intensive Care Unit**	**12 (35.29%)**

Note: SD, standard deviation; SARS-CoV-2, Severe Acute Respiratory Syndrome Coronavirus 2; LVEF, left ventricular ejection fraction.

**Table 2:** Comparison between children with mild and severe MIS-C.

Clinical Parameters and Laboratory Tests (Normal Range)	MIS-C (*n* = 34)	MIS-C Severity		
		Mild Form (*n* = 15, 44.11%)	Severe Form (*n* = 19, 55.88%)	*p*-Value
**Mean Age (years ±SD)**	6.8 ± 4.6	6.7 ± 10.3	5.3 ± 2.08	0.554111
**Male (No., %)**	21 (61.76%)	8 (53.33%)	13 (68.42%)	0.3758
**The period between the onset of symptoms and the first day of hospitalization (days ± SD)**	6.7 ± 7.2	8.4 ± 10.5	5.3 ± 2.1	0.219357
**Duration of hospitalization (days ± SD)**	11.6 ± 8.0	**6.9 ± 2.6**	**15.3 ± 8.9**	**0.001202**
**Growth (Z-score ± SD)**	−0.5 ± 2.1	−1.0 ± 2.8	−0.1 ± 1.3	0.222802
**Duration of fever (days ± SD)**		**4.3 ± 2.9**	**7.5 ± 2.8**	**0.003500**
**CBC** WBC (4.2–9.4×10^3^/μL) Hb (10–13 g/dL) P (194–345×10^3^/μL)	11.440.2 ± 5578.6 11.5 ± 1.7 253.147.3 ± 162.799.0	12.155 ± 5.538 12.1 ± 0.0 **347.800 ± 162.062**	10.875 ± 5.694 11.1 ± 2.0 **178.421 ± 121.662**	0.514972 0.106651 **0.001456**
**Inflammatory markers** ESR (0–20 mm/h) ESR max (mm/h)	34.6 ± 19.6	36.2 ± 20.4 43.8 ± 18.1	33.3 ± 19.4 50.6 ± 25.8	0.671182 0.391620
CRP (0–1 mg/L) CRP max (mg/L)	12.1 ± 7.4	**8.2 ± 6.0** **10.0 ± 5.8**	**15.1 ± 7.0** **16.8 ± 6.1**	**0.004433** **0.002503**
Procalcitonin (<0.8 g/dL) Procalcitonin max (g/dL)	6.3 ± 15.6	1.7 ± 2.7 1.9 ± 2.8	10.0 ± 20.2 11.7 ± 20.1	0.122764 0.072844
Ferritin (<300 ng/mL) LDH (120–330 g/L) (*n* = 29) IL-6 (<5 pg/mL) (*n* = 25)	763.7 ± 2274.9 24 ± 334.5	179.1 ± 270.9 259.9 ± 64.2 33.7 ± 22.9	1227.1 ± 2986.5 380.5 ± 277.4 214.8 ± 385.8	0.186437 0.122081 0.176261
**Hepatic function** AST (10–37 U/L) ALT (9–40 U/L) Albumin (3.9–5.2 g/dL)	102.5 ± 325.5 100.5 ± 358.7 3.4 ± 0.6	45.3 ± 36.5 37.9 ± 42.3 **3.8 ± 0.5**	147.6 ± 433.9 150.0 ± 478.2 **3.1 ± 0.5**	0.370580 0.373564 **0.001963**
**Renal function** Urea (5–18 mg/dL) Creatinine (<1.0 mg/dL)	27.6 ± 13.1 0.4 ± 0.2	23.3 ± 8.5 0.4 ± 0.2	31.0 ± 15.2 0.5 ± 0.3	0.086228 0.316754
**Coagulopathy** Fibrinogen (200–400 mg/dL) D-dimers (250 ng/mL)	429.3 ± 129.0 2488.4 ± 3459.0	460.4 ± 124.5 1389.9 ± 1359.4	404.5 ± 130.3 3297.9 ± 4278.5	0.224362 0.118825
**Cardiac markers** NT-proBNP (<125 pg/mL) Troponin (<35 ng/L)	3998.9 ± 7231.7 18 ± 27.9	**288.2 ± 416.9** **2.6 ± 0.4**	**6733.2 ± 8.616.6** **28.6 ± 32.4**	**0.009057** **0.007231**
**Other** CK (8–147 U/L) TGL (<150 mg/dL)	52 ± 38 173.6 ± 114.7	49.4 ± 30.6 **116.3 ± 56.4**	53.8 ± 43.0 **218.8 ± 129.5**	0.764305 **0.007515**
**Glycemia, electrolytes** Glucose (60–99 mg/dL) Na (134–143 mEq/L) K (3.4–4.7 mEq/L)	105.7 ± 42.9 135.9 ± 4.1 4.1 ± 0.5	91.9 ± 16.6 **137.9 ± 2.9** 4.3 ± 0.4	116 ± 53.6 **134.3 ± 4.3** 4.0 ± 0.6	0.095998 **0.008941** 0.067727

Note: CBC, complete blood count; WBC, white blood cells; Hb, hemoglobin; P, platelet count; ESR, erythrocyte sedimentation rate; CRP, C-reactive protein; LDH, lactate dehydrogenase; IL-6, interleukin-6; AST, aspartate aminotransferase; ALT, alanine aminotransferase; NT-proBNP, brain natriuretic peptide; CK, creatine kinase; TGL, triglycerides; Na, sodium; K, potassium. The results are presented as mean and standard deviation or number and percentage (for gender). The *p*-value represents the statistical significance of the differences between different parameters in mild and severe forms (*p* < 0.05 in bold).

**Table 3:** Organ and tissues injuries in MIS-C.

Cohort Characteristics (*n* = 34 Cases)		Mild Disease Form (*n* = 15 Cases)	Severe Disease Form (*n* = 19 Cases)	*p*-Value
Multiorgan Disfunction *	**Organ Disfunction Number** 2–3 organs 4–5 organs 6–8 organs >8 organs	**9 (60%)** 6 (40%) **0** 0	**1 (5.26%)** 4 (21.06%) **10 (52.64%)** 4 (21.06%)	**0.0026** 0.2358 **0.0010** 0.0623
**Laboratory parameters impairment**	**Inflammatory markers** Increased ESR (>20 mm/h) Increased CRP (>1 mg/L) Increased procalcitonin (>1 g/dL) Increased Ferritin (>300 ng/mL) Increased LDH (>330 g/L) Increased IL-6 (>5 pg/mL)	**11 (73.34%)** 13 (86.67%) **3 (20%)** **1 (6.67%)** 15 (100%) 7 (*n* = 9; 77.77%)	**19 (100%)** 19 (100%) **19 (100%)** **13 (68.43%)** 19 (100%) 16 (*n* = 16; 100%)	**0.0183** 0.1061 **<0.0001** **0.0003** **-** 0.0540
	**Hepatic dysfunction** Increased AST (>45 U/L) Increased ALT (>50 U/L) Hypoalbuminemia (<3 g/dL)	2 (13.34%) 2 (13.34%) **3 (20%)**	5 (26.32%) 5 (26.32%) **15 (78.95%)**	0.3599 0.3599 **0.0008**
	**Renal dysfunction** Increased Urea (>25 mg/dL) Increased Creatinine (>1.0 mg/dL)	12 (80%) 0	18 (94.74%) 3 (15.79%)	0.1919 0.1123
	**Coagulopathy** Increase Fibrinogen (>400 mg/dL) Increased D-dimers (>250 ng/mL)	9 (60%) **10 (66.67%)**	12 (63.16%) **19 (100%)**	0.8529 **0.0073**
	**Cardiac dysfunction** Increased NT-proBNP (>150 pg/mL) Increased Troponin (>35 ng/L)	**9 (60%)** **0**	**19 (100%)** **7 (36.85%)**	**0.0028** **0.0093**
	**Other** Increased CK (>200 U/L) Increased TGL (>150 mg/dL)	0 **3 (20%)**	1 (5.26%) **13 (68.43%)**	0.3744 **0.0056**
	**Glycemia, electrolytes** Hypoglycemia (<60 mg/dL) Hyponatremia (<130 mEq/L) Hypernatremia (>145 mEq/L) Hypokalemia (<3 mEq/L) Hyperkalaemia (>5 mEq/L)	0 **0** 0 0 5 (33.34%)	0 **7 (36.85%)** 0 3 (15.79%) 11 (57.89%)	- **0.0093** - 0.1123 0.1605
**Long-term evolution**	**Completely recovery** **Partial recovery (chronic complications)** **Death**	**15 (100%)** **0** 0	**12 (63.16%)** **7(36.85%)** 1 (5.26%)	**0.0094** **0.0093** 0.7020

* Multiorgan Dysfunction: gastrointestinal, cardiac, hematological, respiratory, neurological, renal, osteoarticular, cutaneous, and mucous manifestations. Note: ESR, erythrocyte sedimentation rate; CRP, C-reactive protein; LDH, lactate dehydrogenase; IL-6, interleukin-6; AST, aspartate aminotransferase; ALT, alanine aminotransferase; NT-proBNP, brain natriuretic peptide; CK, creatine kinase; TGL, triglycerides. The results are presented as numbers and percentages. The *p*-value represents the statistical significance of the differences between different parameters in mild and severe forms (*p* < 0.05 in bold).

## Data Availability

Not applicable.

## References

[1] Dong Y., Mo X., Hu Y., Qi X., Jiang F., Jiang Z., Tong S. (2020). Epidemiology of COVID-19 Among Children in China. Paediatrics.

[2] Sancho-Shimizu V., Brodin P., Cobat A., Biggs M.C., Toubiana J., Lucas L.C., Henrickson E.S., Belot A., Tangye G.S., Milner D.J. (2021). SARS-CoV-2–related MIS-C: A key to the viral and genetic causes of Kawasaki disease?. J. Exp. Med..

[3] Diorio C., Henrickson S.E., Vella L.A., McNerney K.O., Chase J., Burudpakdee C., Lee J.H., Jasen C., Balamuth F., Barrett D.M. (2020). Multisystem inflammatory syndrome in children and COVID-19 are distinct presentations of SARS-CoV-2. J. Clin. Investig..

[4] Abrams J.Y., Godfred-Cato S.E., Oster M.E., Chow E.J., Koumans E.H., Bryant B., Leung J.W., Belay E.D. (2020). Multisystem Inflammatory Syndrome in Children Associated with Severe Acute Respiratory Syndrome Coronavirus 2: A Systematic Review. J. Pediatr..

[5] Cheng M.H., Zhang S., Porritt R.A., Noval Rivas M., Paschold L., Willscher E., Binder M., Arditi M., Bahar I. (2020). Superantigenic character of an insert unique to SARS-CoV-2 spike supported by skewed TCR repertoire in patients with hyperinflammation. Proc. Natl. Acad. Sci. USA.

[6] Boneparth A., Kernie S.G., Orange J.S., Milner J.D. (2020). Multisystem Inflammatory Syndrome Related to COVID-19 in Previously Healthy Children and Adolescents in New York City. JAMA.

[7] Khemani R.G., Smith L., Lopez-Fernandez Y.M., Kwok J., Morzov R., Klein M.J., Yehya N., Willson D., Kneyber M., Lillie J. (2019). Pediatric Acute Respiratory Distress Syndrome Incidence and Epidemiology (PARDIE) Investigators, & Pediatric Acute Lung Injury and Sepsis Investigators (PALISI) Network. Paediatric acute respiratory distress syndrome incidence and epidemiology (PARDIE): An international, observational study. Lancet Respir. Med..

[8] Belhadjer Z., Méot M., Bajolle F., Khraiche D., Legendre A., Abakka S., Auriau J., Grimaud M., Oualha M., Beghetti M. (2020). Acute Heart Failure in Multisystem Inflammatory Syndrome in Children in the Context of Global SARS-CoV-2 Pandemic. Circulation.

[9] Cho M.H. (2020). Pediatric Acute Kidney Injury: Focusing on Diagnosis and Management. Kidney Dis..

[10] Alonso E., Squires R., Whitington P., Suchy F., Sokol R., Balistreri W. (2007). Acute Liver Failure in Children. Liver Disease in Children.

[11] Squires R.H. (2008). Acute liver failure in children. Semin. Liver Dis..

[12] Grama A., Burac L., Aldea C.O., Bulata B., Delean D., Samasca G., Abrudan C., Sirbe C., Pop T.L. (2020). Vitamin D-Binding Protein (Gc-Globulin) in Acute Liver Failure in Children. Diagnostics.

[13] O’Loughlin L., Alvarez Toledo N., Budrie L., Waechter R., Rayner J. (2021). A Systematic Review of Severe Neurological Manifestations in Pediatric Patients with Coexisting SARS-CoV-2 Infection. Neurol. Int..

[14] Henter J.-I., Horne A., Aricó M., Egeler R.M., Filipovich A.H., Imashuku S., Ladisch S., McClain K., Webb D., Winiarski J. (2007). HLH-2004: Diagnostic and therapeutic guidelines for hemophagocytic lymphohistiocytosis. Pediatr. Blood Cancer.

[15] Miller A.D., Zambrano L.D., Yousaf A.R., Abrams J.Y., Meng L., Wu M.J., Melgar M., Oster M.E., Godfred Cato S.E., Belay E.D. (2021). Multisystem Inflammatory Syndrome in Children—United States, February 2020–July 2021. Clin. Infect. Dis..

[16] Tran V.L., Parsons S., Nuibe A. (2021). The Trilogy of SARS-CoV-2 in Pediatrics (Part 2): Multisystem Inflammatory Syndrome in Children. J. Pediatr. Pharmacol. Ther. JPPT Off. J. PPAG.

[17] Algarni A.S., Alamri N.M., Khayat N.Z., Alabdali R.A., Alsubhi R.S., Alghamdi S.H. (2022). Clinical practice guidelines in multisystem inflammatory syndrome (MIS-C) related to COVID-19: A critical review and recommendations. World J. Pediatr. WJP.

[18] Lazova S., Alexandrova T., Gorelyova-Stefanova N., Atanasov K., Tzotcheva I., Velikova T. (2021). Liver Involvement in Children with COVID-19 and Multisystem Inflammatory Syndrome: A Single-Center Bulgarian Observational Study. Microorganisms.

[19] Nardo A.D., Schneeweiss-Gleixner M., Bakail M., Dixon E.D., Lax S.F., Trauner M. (2021). Pathophysiological mechanisms of liver injury in COVID-19. Liver Int. Off. J. Int. Assoc. Study Liver.

[20] Radzina M., Putrins D.S., Micena A., Vanaga I., Kolesova O., Platkajis A., Viksna L. (2022). Post-COVID-19 Liver Injury: Comprehensive Imaging with Multiparametric Ultrasound. J. Ultrasound Med. Off. J. Am. Inst. Ultrasound Med..

[21] Cantor A., Miller J., Zachariah P., DaSilva B., Margolis K., Martinez M. (2022). Acute Hepatitis Is a Prominent Presentation of the Multisystem Inflammatory Syndrome in Children: A Single-Center Report. Hepatology.

[22] Friedman K.G., Harrild D.M., Newburger J.W. (2020). Cardiac Dysfunction in Multisystem Inflammatory Syndrome in Children: A Call to Action. J. Am. Coll. Cardiol..

[23] Okarska-Napierała M., Zalewska E., Kuchar E. (2021). Fever and Diarrhea as the Only Symptoms of Multisystem Inflammatory Syndrome in Children. Gastroenterology.

[24] Farias E., Justino M., Mello M. (2020). Multisystem Inflammatory Syndrome in A Child Associated with Coronavirus Disease 19 In the Brazilian Amazon: Fatal Outcome in An Infant. Rev. Paul. Pediatr. Orgao Of. Soc. Pediatr. Sao Paulo.

[25] Ciftdogan D.Y., Keles Y.E., Karbuz A., Cetin B.S., Bozdemir S.E., Kadayifci E.K., Akcan O.M., Ozer A., Erat T., Sutcu M. (2022). Multisystem inflammatory syndrome in children associated with COVID-19 in 101 cases from Turkey (Turk-MISC study). J. Paediatr. Child Health.

[26] Ko R., Massa C., Saraiya N., Cheung E.W. (2022). Multisystem Inflammatory Syndrome in Children (MIS-C): Experiences with a New Disease Process. J. Neurosurg. Anesthesiol..

[27] Dufort E.M., Koumans E.H., Chow E.J., Rosenthal E.M., Muse A., Rowlands J., Barranco M.A., Maxted A.M., Rosenberg E.S., Easton D. (2020). New York State and Centers for Disease Control and Prevention Multisystem Inflammatory Syndrome in Children Investigation Team. Multisystem Inflammatory Syndrome in Children in New York State. N. Engl. J. Med..

[28] Ahmed M., Advani S., Moreira A., Zoretic S., Martinez J., Chorath K., Acosta S., Naqvi R., Burmeister-Morton F., Burmeister F. (2020). Multisystem inflammatory syndrome in children: A systematic review. EClinicalMedicine.

[29] Davies P. (2021). Addressing fundamental questions on MIS-C. The Lancet. Child Adolesc. Health.

[30] Godfred-Cato S., Tsang C.A., Giovanni J., Abrams J., Oster M.E., Lee E.H., Lash M.K., Le Marchand C., Liu C.Y., Newhouse C.N. (2021). Multisystem Inflammatory Syndrome in Infants <12 months of Age, United States, May 2020–January 2021. Pediatr. Infect. Dis. J..

[31] Alsaied T., Tremoulet A.H., Burns J.C., Saidi A., Dionne A., Lang S.M., Newburger J.W., de Ferranti S., Friedman K.G. (2021). Review of Cardiac Involvement in Multisystem Inflammatory Syndrome in Children. Circulation.

[32] Blondiaux E., Parisot P., Redheuil A., Tzaroukian L., Levy Y., Sileo C., Schnuriger A., Lorrot M., Guedj R., Ducou le Pointe H. (2020). Cardiac MRI in Children with Multisystem Inflammatory Syndrome Associated with COVID-19. Radiology.

[33] McMurray J.C., May J.W., Cunningham M.W., Jones O.Y. (2020). Multisystem Inflammatory Syndrome in Children (MIS-C), a Post-viral Myocarditis and Systemic Vasculitis-A Critical Review of Its Pathogenesis and Treatment. Front. Pediatr..

[34] Vagrecha A., Zhang M., Acharya S., Lozinsky S., Singer A., Levine C., Al-Ghafry M., Fein Levy C., Cron R.Q. (2022). Hemophagocytic Lymphohistiocytosis Gene Variants in Multisystem Inflammatory Syndrome in Children. Biology.

[35] Menon N.M., Srivaths L.V. (2022). Thromboembolism in children with multisystem inflammatory syndrome: A literature review. Pediatr. Res..

[36] Naka F., Melnick L., Gorelik M., Morel K.D. (2021). A dermatologic perspective on multisystem inflammatory syndrome in children. Clin. Dermatol..

[37] Marzano A.V., Cassano N., Moltrasio C., Verdoni L., Genovese G., Vena G.A. (2021). Multisystem Inflammatory Syndrome in Children Associated with COVID-19: A Review with an Emphasis on Mucocutaneous and Kawasaki Disease-Like Findings. Dermatology.

[38] Olivotto S.B.E. (2021). Acute encephalitis in pediatric multisystem inflammatory syndrome associated with COVID-19. Eur. J. Paediatr. Neurol..

[39] Abdel-Mannan O., Eyre M., Löbel U., Bamford A., Eltze C., Hameed B., Hemingway C., Hacohen Y. (2020). Neurologic and Radiographic Findings Associated With COVID-19 Infection in Children. JAMA Neurol..

[40] Henderson L.A., Canna S.W., Friedman K.G., Gorelik M., Lapidus S.K., Bassiri H., Behrens E.M., Ferris A., Kernan K.F., Schulert G.S. (2021). American College of Rheumatology Clinical Guidance for Multisystem Inflammatory Syndrome in Children Associated With SARS-CoV-2 and Hyperinflammation in Pediatric COVID-19: Version 1. Arthritis Rheumatol..

